# Mycophenolate Mofetil for Steroid-Refractory Immune-Related Hepatitis and Sclerosing Cholangitis Following Neoadjuvant Chemoimmunotherapy in Non-Small Cell Lung Cancer: A Case Report

**DOI:** 10.70352/scrj.cr.25-0192

**Published:** 2025-05-21

**Authors:** Hideto Iguchi, Takahiro Kaki, Yuhei Harutani, Daiki Kitahara, Yoshimitsu Hirai, Kuninobu Kanai, Issei Hirai

**Affiliations:** 1 Department of Breast and General Thoracic Surgery, Naga Municipal Hospital, Kinokawa, Wakayama, Japan; 2 Department of Thoracic and Cardiovascular Surgery, Wakayama Medical University, Wakayama, Wakayama, Japan; 3 Department of Respiratory Medicine, Naga Municipal Hospital, Kinokawa, Wakayama, Japan

**Keywords:** immune-related adverse event, mycophenolate mofetil, neoadjuvant chemoimmunotherapy, non-small cell lung cancer, bronchial fistula

## Abstract

**INTRODUCTION:**

Neoadjuvant chemoimmunotherapy is increasingly regarded as the standard treatment for resectable non-small cell lung cancer. Although it improves survival outcomes, immune-related adverse events can delay or prevent curative surgery. Optimal strategies for managing these adverse events in the preoperative setting remain unclear. This case is notable for being, to the best of our knowledge, the first to report curative surgery following treatment of neoadjuvant chemoimmunotherapy-induced, steroid-refractory immune-related hepatitis using mycophenolate mofetil.

**CASE PRESENTATION:**

A 74-year-old man with stage IIIA (cT2bN2M0) squamous cell carcinoma of the right lower lobe received neoadjuvant chemoimmunotherapy consisting of carboplatin, paclitaxel, and nivolumab. Following 2 treatment cycles, he developed fever, jaundice, and grade 3 liver dysfunction. Laboratory and imaging studies revealed features consistent with hepatitis and sclerosing cholangitis, suspected to be immune-related. High-dose corticosteroids were administered, resulting in only transient improvement. Owing to steroid-refractory disease, mycophenolate mofetil was initiated, leading to normalization of liver function and resolution of symptoms. However, the primary tumor exhibited regrowth following immunosuppression. Surgical resection was performed, consisting of right middle and lower lobectomy with lymph node dissection. Histopathology confirmed ypT1cN0M0 stage IA3 with 50% residual viable tumor. The postoperative course was complicated by persistent air leakage, empyema, and a bronchial fistula, ultimately requiring open-window thoracostomy. The patient was discharged and remains free of disease recurrence at follow-up.

**CONCLUSIONS:**

This case highlights the potential role of mycophenolate mofetil in managing steroid-refractory immune-related liver injury induced by neoadjuvant chemoimmunotherapy in non-small cell lung cancer. Although immunosuppressive therapy may enable definitive surgery, it may also contribute to tumor regrowth and serious postoperative complications. As the use of neoadjuvant chemoimmunotherapy expands, further clinical experience is needed to guide the management of immune-related adverse events and ensure safe and effective surgical outcomes.

## Abbreviations

AE
: adverse event
ALK
: anaplastic lymphoma kinase
AUC
: area under the curve
EGFR
: epidermal growth factor
ICI
: immune checkpoint inhibitor
irAE
: immune-related adverse event
MMF
: mycophenolate mofetil
NSCLC
: non-small cell lung cancer
PD-L1
: programmed cell death ligand 1
RECIST
: Response Evaluation Criteria in Solid Tumors

## INTRODUCTION

Neoadjuvant chemoimmunotherapy has become the standard of care for patients with resectable, early-stage NSCLC.^1)^ However, serious AEs remain a major clinical concern, as they can result in surgical delays or cancellations. Clinical trials have reported AE-related surgical delays in approximately 20% of cases and cancellations in approximately 1%.^1)^ Among these, irAEs require particular attention. Although appropriate preoperative management of irAEs is crucial, detailed guidance on optimal treatment strategies remains limited. Here, we report a case of neoadjuvant chemoimmunotherapy-induced, steroid-refractory, irAE-related liver dysfunction that improved with MMF treatment, enabling curative surgery. This is the first documented case in which neoadjuvant chemoimmunotherapy-induced irAE hepatitis was successfully managed with MMF, enabling definitive surgical resection.

## CASE PRESENTATION

A 74-year-old man with a history of hypertension and chronic kidney disease, and a performance status of 1 was diagnosed with squamous cell lung carcinoma of the right lower lobe. The clinical stage was IIIA (T2bN2M0) based on the Union for International Cancer Control TNM classification, version 8, with involvement of station 7 lymph nodes. Molecular profiling showed EGFR wild-type and ALK-negative status, and PD-L1 expression was 35% (PD-L1 IHC 22C3 pharmDx [Dako]). The patient received neoadjuvant chemoimmunotherapy with carboplatin (AUC = 6), paclitaxel (200 mg/m^2^), and nivolumab (360 mg).

**Fig. 1:**
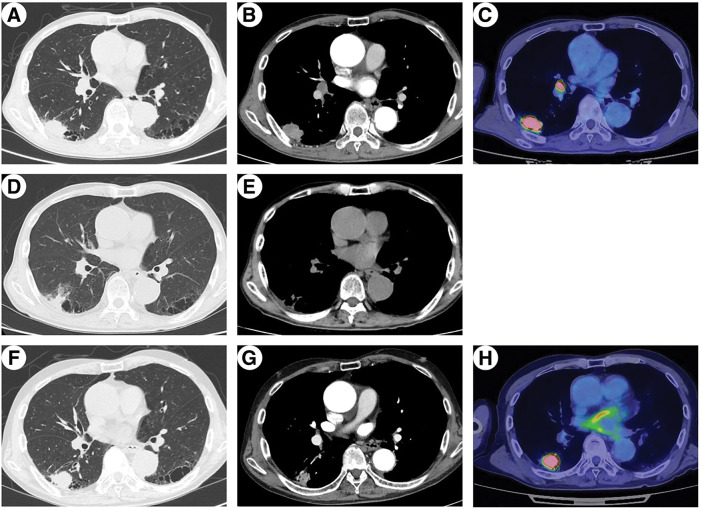
Chest CT and FDG-PET. Images demonstrate tumor response to neoadjuvant chemoimmunotherapy and subsequent regrowth. (**A**–**C**) Pre-neoadjuvant chemoimmunotherapy chest CT/FDG-PET showing a 42-mm right lung tumor and FDG uptake in the No.11i lymph node. (**D**, **E**) Chest CT before steroid treatment showing tumor shrinkage (19 mm) and reduced No.11i lymph node. (**F**–**H**) Preoperative chest CT showing regrowth of tumor (33 mm), whereas No.11i lymph node remained reduced in size, making preoperative biopsy difficult. FDG, fluorodeoxyglucose

**Fig. 2:**
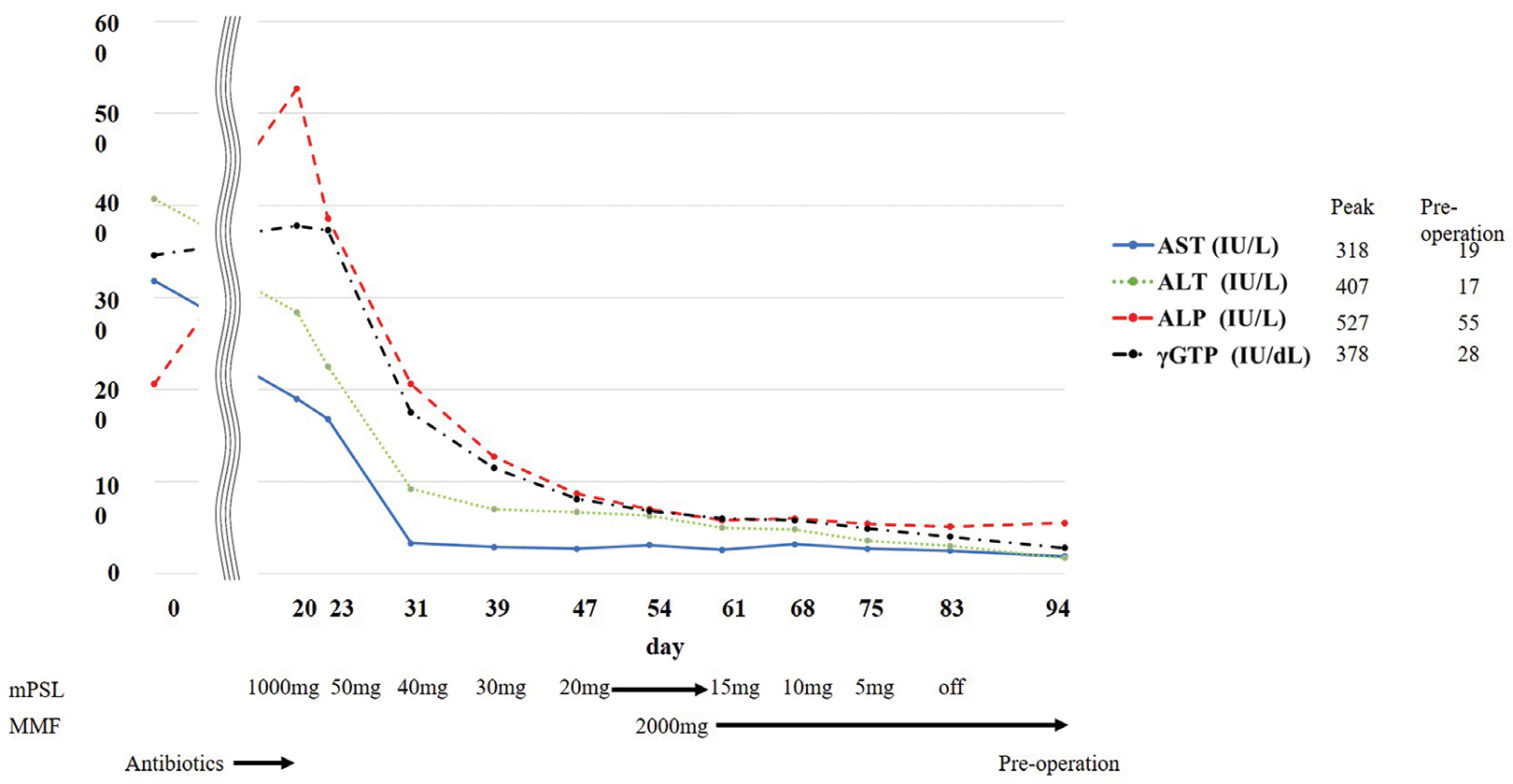
Clinical course of liver dysfunction and treatment timeline. Steroid therapy was initiated on day 20, resulting in initial improvement in enzyme levels. Due to tapering difficulties, MMF was administered on day 54, after which steroid tapering was successfully completed. Surgery was performed on day 95. MMF, mycophenolate mofetil

After 2 cycles, the patient presented to the emergency department with fever and jaundice. RECIST 1.1 indicated a partial response, with tumor size decreasing from 42 to 19 mm on chest CT (**Fig. 1A** and **1D**). Blood tests revealed elevated levels of inflammatory markers and grade 3 liver dysfunction (Common Terminology Criteria for Adverse Events version 5.0) (**Fig. 2**, Day 0). Viral hepatitis markers, antinuclear antibodies, and anti-mitochondrial antibodies were negative. Abdominal CT revealed bile sludge, raising suspicion for cholecystitis or cholangitis. However, antibiotics were ineffective. Thus, additional diagnostic tests were performed. Magnetic resonance cholangiopancreatography revealed diffuse biliary duct stenosis and sclerosis without ductal obstruction. Liver biopsy revealed mild CD8-positive T cell infiltration and periportal fibrosis. Consultation with a hepatology specialist indicated that the findings were consistent with irAE-induced hepatitis and sclerosing cholangitis.

Corticosteroid therapy with intravenous methylprednisolone (1000 mg/day) was initiated on day 20. Although liver dysfunction initially improved with steroid therapy, the effect was insufficient and further steroid tapering was difficult. After a multidisciplinary team conference, MMF was initiated for the management of irAE hepatitis, with informed consent obtained regarding its off-label use. Therefore, MMF (2000 mg/day) was introduced on day 54, leading to symptom resolution, improvement in liver enzyme levels, and successful steroid tapering. However, the primary tumor increased in size from 19 to 33 mm, as observed on chest CT (**Fig. 1D** and **1F**), meeting RECIST criteria for progressive disease. A multidisciplinary team conference concluded that early surgical resection was appropriate to achieve cure given the tumor re-progression. To minimize perioperative complications, surgery was scheduled after completion of steroid therapy. Surgery was performed on day 95.

Due to infiltration of the No.11i lymph node, dissection from the pulmonary artery was not feasible; hence, right middle and lower lobectomy with ND2a-2 lymph node dissection was performed. Postoperative pathological evaluation revealed ypT1cN0M0 stage IA3 with 50% residual viable tumor. Postoperatively, persistent air leakage required talc pleurodesis. After confirming cessation of air leakage, the chest drain was removed on postoperative day 12. However, on postoperative day 15, empyema developed, requiring repeated thoracic drainage. Recurrent air leakage led to bronchial fistula diagnosis via bronchoscopy on postoperative day 26, so emergency open-window thoracostomy was performed. MMF was discontinued on postoperative day 35 as no re-elevation of liver enzymes was observed and the irAE hepatitis was deemed resolved. The patient was discharged 106 days after the initial surgery and remains under outpatient follow-up with an open wound, maintaining good general health and showing no evidence of recurrent liver dysfunction or lung cancer about 5 months postoperatively.

## DISCUSSION

We encountered a case of neoadjuvant chemoimmunotherapy-induced, steroid-refractory irAE hepatitis effectively managed with MMF, enabling definitive surgical resection. Although MMF improved hepatic dysfunction, its use may have contributed to tumor regrowth and postoperative complications.

ICIs cause hepatitis in 5%–10% of patients (grade ≥3 in 1%–2%),^2)^ and steroid-refractory irAE hepatitis occurs in 12%–48% of patients.^3,4)^ Guidelines recommend MMF for steroid-refractory irAE hepatitis,^2)^ and several reports support its efficacy.^3,4)^ However, evidence on managing irAEs during neoadjuvant chemoimmunotherapy is scarce. To our knowledge, this is the first report showing that MMF can effectively treat neoadjuvant chemoimmunotherapy‑induced irAE hepatitis in NSCLC and facilitate curative surgery.

Although immunosuppressive therapy is effective, it may also promote tumor progression.^5)^ Ogusu et al. reported shorter progression-free survival after immunosuppression.^6)^ In our case, tumor regrowth occurred following MMF administration, underscoring the importance of frequent imaging to monitor tumor progression during irAE treatment.

Bronchial fistula developed postoperatively. The bronchial artery was successfully preserved, and no significant intraoperative issues were noted, suggesting that the complication was not due to the surgical technique. Reported risk factors for bronchial fistulas following lung cancer surgery include preoperative chemotherapy, bronchial ischemia, immunosuppression, and infection.^7,8)^ McAnally et al. found higher bronchial complication rates with high-dose steroid use in lung transplantation as compared with low-dose steroid administration.^9)^ No studies have explored the potential relationship between corticosteroids and MMF in the treatment of irAEs following neoadjuvant chemoimmunotherapy and the risk of bronchial fistulas. Although the exact cause of the bronchial fistula was unclear, steroid and MMF use may have contributed. Consequently, in cases where steroid-based treatments are necessary to manage irAEs following neoadjuvant chemoimmunotherapy, it is important to maintain heightened vigilance for serious postoperative complications, including bronchial stump fistula. With the expected rise in the use of neoadjuvant chemoimmunotherapy for NSCLC, the accumulation of additional cases will be essential to enhance the prediction of postoperative complication risks and to establish optimal strategies for the management of irAEs.

## CONCLUSIONS

Although MMF is effective in managing steroid-refractory irAE-related liver injury, its immunosuppressive effects may contribute to tumor progression and increase the risk of postoperative complications. Careful monitoring and multidisciplinary management of irAEs are essential during the preoperative period to ensure surgical eligibility and optimize patient outcomes.

## ACKNOWLEDGMENTS

We would like to thank Editage (www.editage.jp) for English language editing.

## DECLARATIONS

### Funding

Not applicable.

### Authors’ contributions

HI: conceptualization, data curation, investigation, operational performance, and writing of the original draft.

TK and Y Harutani: treatment and investigation.

KD and KK: treatment.

Y Hirai: operational performance.

IH: operational performance, treatment and supervision.

All authors reviewed and approved the final version of the manuscript and had accountability for all aspects of the work.

### Availability of data and materials

Not applicable.

### Ethics approval and consent to participate

This work does not require ethical considerations or approval. Informed consent was obtained for this case report.

### Consent for publication

Written informed consent was obtained from the patient for the publication of this case report and any accompanying images.

### Competing interests

The authors declare that they have no competing interests.
